# Using mechanistic Bayesian networks to identify downstream targets of the Sonic Hedgehog pathway

**DOI:** 10.1186/1471-2105-10-433

**Published:** 2009-12-18

**Authors:** Abhik Shah, Toyoaki Tenzen, Andrew P McMahon, Peter J Woolf

**Affiliations:** 1 Bioinformatics Program, University of Michigan, Ann Arbor, MI 48109, USA; 2 Stowers Medical Institute, Center for Regenerative Medicine, Cardiovascular Research Center, Massachusetts General Hospital, Boston, MA 02114, USA; 3 Harvard Stem Cell Institute, Boston, MA 02114, USA; 4 Department of Stem Cell and Regenerative Biology, Harvard University, Cambridge MA 02138, USA; 5 Department of Molecular and Cellular Biology, Harvard University, Cambridge MA, 02138, USA; 6 Department of Biomedical Engineering, University of Michigan, Ann Arbor, MI 48109, USA; 7 Department of Chemical Engineering, University of Michigan, Ann Arbor, MI 48109, USA

## Abstract

**Background:**

The topology of a biological pathway provides clues as to how a pathway operates, but rationally using this topology information with observed gene expression data remains a challenge.

**Results:**

We introduce a new general-purpose analytic method called Mechanistic Bayesian Networks (MBNs) that allows for the integration of gene expression data and known constraints within a signal or regulatory pathway to predict new downstream pathway targets. The MBN framework is implemented in an open-source Bayesian network learning package, the Python Environment for Bayesian Learning (PEBL). We demonstrate how MBNs can be used by modeling the early steps of the sonic hedgehog pathway using gene expression data from different developmental stages and genetic backgrounds in mouse. Using the MBN approach we are able to automatically identify many of the known downstream targets of the hedgehog pathway such as *Gas1 *and *Gli1*, along with a short list of likely targets such as *Mig12*.

**Conclusions:**

The MBN approach shown here can easily be extended to other pathways and data types to yield a more mechanistic framework for learning genetic regulatory models.

## Background

A general problem in systems biology is the integration of observational experimental data such as gene expression, with known pathways topologies. Ideally these two data sources should be complementary, however in practice there are few methods to systematically integrate these two kinds of information. In this paper, we introduce a method called Mechanistic Bayesian Networks (MBN) in an attempt to use knowledge about the topology of a pathway with gene expression data related to the same pathway. As a sample case we used the topology of the Sonic Hedgehog (*Shh*) signaling pathway as a model pathway along with a targeted gene expression dataset. Using these data we used the MBN approach to identify regulatory targets of the Shh pathway.

### Sonic Hedgehog (Shh) pathway

**Figure 1:**
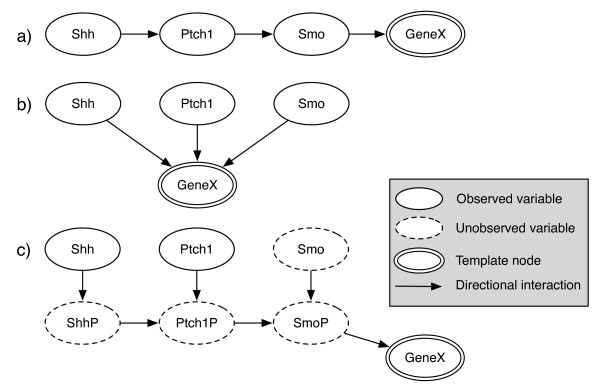
**BN and MBN modeling of Shh pathway**: (a) A sequential BN model of the pathway that omits protein activity, (b) a parallel BN model of the pathway, and (c) an MBN model of the pathway. The ovals *Shh*, *Ptch1 *and *Smo *represent mRNA measurements, while the ovals, ShhP, Ptch1P and SmoP, represent proteins. The oval GeneX indicates a candidate downstream expression target of the pathway. Dotted ovals represent entities that are known in the mechanism but are not observed, while unbroken ovals represent experimentally observed variables. Arrows between nodes represent a directional interaction but do not specify the functional form.

The Shh pathway plays a central role in organismal development and the progression of some cancers [1]. Because of its central role, the Shh pathway is well studied providing us with an ideal test case to validate our MBN approach. The details of Shh are reviewed in detail elsewhere [2-4], but here we will summarize the early steps of the pathway that we will use in this work. *Shh *is a secreted protein that acts as both a short-range contact-dependant factor and as a long-range diffusible morphogen. The Shh ligand binds its canonical receptor Patched1 (*Ptch1*), which releases its inhibition of a second membrane-bound protein, Smoothened (*Smo*). Derepression of *Smo *in turn activates a signaling cascade inside the cell, eventually activating the Gli transcription factors and regulating the expression of a variety of genes. While some of the downstream targets of the pathway are known, many remain unknown. The initial steps of the pathway are shown in Figure 1(c) with the terminal node GeneX representing a putative downstream target. Note that in this cascade, the *Shh*, *Ptch1*, and *Smo *proteins can all directly or indirectly affect the target, although in different ways and to different degrees. Figure 1(a) and 1(b) show more abstracted representations of the pathway.

Due to its dose-dependant effect and role in development, the Shh signal requires strict spatiotemporal control. The pathway's known targets include pathway components, promoters and antagonists, thus regulating the effects of the Shh ligand over time. Cell surface proteins that promote Shh signaling, for example, are initially expressed in Shh-responsive cells, sensitizing cells to even low levels of the Shh ligand. As the level of Shh signaling increases, downregulation of positive Shh components such as *Gas1 *and upregulation of negative components such as *Ptch1*, *Ptch2*, and *Hhip *that sequester the ligand ensure a tight control over Shh signaling [5]. Additionally, *Shh *is known to cooperate with or antagonize other pathways such as Bmp [6], retinoic acid [7,8], Wnt [9], Ras [10], and Notch [11] in a time-, dose-, and spatially-dependant manner. These factors further complicate the identification of pathway targets.

The Shh pathway raises a more general problem: given gene expression data from multiple samples, tissue and organs under different genetic knockout conditions, how can we identify downstream targets of a given pathway? Due to the interactions between different pathways and multiple cascades between ligand reception and eventual transcriptional regulation, it is not clear what constitutes a downstream target of a specific pathway. Common analysis approaches include significance testing between samples, differential expression, and clustering [12]. While these approaches are widely used and helpful, they fail to incorporate knowledge of the underlying pathway. When the pathway information is used, it is done while ignoring fundamental biological knowledge such as the central dogma. DNA, mRNA, and proteins are conflated into one variable and specific interactions such as protein-protein or protein-DNA are implicitly assumed to be detectable via gene expression data. To circumvent this problem, we present a novel mechanistic Bayesian network approach that more closely respects the meaning of both the pathway and the expression data.

## Methods

In the following sections we describe the theoretical underpinnings of the MBN framework and provide a method for evaluating the significance of a result. Next we describe how we tested the MBN method using gene expression data from gene knockout mouse models to identify targets of the Shh pathway.

### Mechanistic Bayesian Networks: Theory and Definition

Mechanistic Bayesian networks represent a subset of a general class of analysis tools called Bayesian Networks (BN). A BN is a probabilistic graphical model that encodes dependencies between variables in a compact and descriptive manner. A BN can be represented as a graph with nodes representing variables and edges representing dependencies or relationships between the variables. Mathematically, each node describes a conditional probability distribution (CPD) that quantitatively models the relationships between a node and its parent nodes. Note that an edge (an arrow) between two variables indicates a directed relationship but does not specify the functional form of the relationship. Said another way, an edge in a Bayesian network does not indicate activation, inhibition or any other specific function. This interpretation of an edge differs from the usual definition of an edge in a signaling pathway, where edges are often assumed to have a defined effect. The advantage of the more broad edge definition is that the relationships between nodes in a Bayesian network can be more complex and include functions that are nonlinear, multimodal, or logical.

BNs have been used successfully to model complex phenomenon in many fields and in systems biology, in particular, to model gene-regulatory networks, protein-interaction networks, signaling networks and to integrate heterogeneous biological data [13-17]. Methods for training and learning BNs are well established [18,19] and are available in a large number of software applications [20].

In contrast to the more general Bayesian network, a Mechanistic Bayesian Network (MBN) adheres more closely to known biological mechanisms by differentiating between mRNA transcripts and proteins, and by including pathway-based structural constraints. Because most studies do not measure both protein and gene expression, unknown quantities in an MBN are treated as unobserved, latent variables. By creating models that more closely resemble the known mechanism, MBNs effectively incorporate in additional information that is not available in the experimental data alone.

### Mechanistic Bayesian Network Templates

When analyzing a biological system, users often want to generate and rank a set of entities that match certain constraints, such as "all downstream targets of the Shh pathway" or "all proteins that participate in the crosstalk between the Shh and Wnt pathways". While it is possible to devise specific sets of constraints and methods for each case, we propose a generic MBN based template approach. Each set of constraints is expressed as an MBN template from which specific MBNs are instantiated and evaluated against an experimental dataset. An MBN template is composed of two types of nodes: constant and template nodes. A constant node is a regular node corresponding to either one variable in the dataset or an unobserved variable while a template node is a holding place for variables in the dataset. Each instantiation of the template is a regular MBN that can be scored using existing BN methods.

### Maximum Entropy Discretization of the data

Before MBN can be applied to a dataset, the data must be discretized into a finite set of bins. While there are many ways to bin data, we use the Maximum Entropy principle to derive our discretization scheme. The principle states that the distribution that maximizes the information entropy is the true distribution given testable information [21].

Accordingly, we bin our data such that each bin contains the same number of data points as this maximizes the entropy of the distribution. There is no theory prescribing the optimal number of bins for any given dataset or analytical method; increasing the number of bin decreases the information loss incurred during discretization but also increases the number of parameters and thus decreases the statistical power of the analysis given the same data samples. Most modelers working with systems biology data have arrived at three bins as a suitable compromise between information loss and statistical power [13,15,22].

### Calculating the Posterior Probability of a Model

Once the data are discretized, a particular model topology can be scored as the posterior probability of a model given data, given below.(1)

P(M|D) is the posterior probability of the BN or MBN model M given the data D. Said another way, P(M|D) is our belief that the model M is correct after having observed the data D. P(M) is the prior probability of the model, that is, our belief that the model M is correct before having observed the data D. The prior probability allows us to integrate model probabilities computed using other methods and data. P(D|M) is the likelihood that the data was generated from the model M while P(D) is a scaling factor that is usually ignored. P(D|M) is calculated as the likelihood after marginalizing over all model parameters given a network structure.

In the MBN framework, a Bayesian network is modeled using a multinomial representation with Dirichlet priors for the relationships between variables. This representation is convenient in that it is relatively agnostic to functional forms and has a convenient closed form solution, the Bayesian Dirichlet Equivalent (BDe) metric, described below.

### BDe Scoring Metric: An approximation for the Marginal Likelihood

The MBN approach uses the BDe scoring metric [19] to calculate the marginal likelihood of a dataset given a model. The BDe metric is a closed form solution to the marginal likelihood for a multinomial Bayesian network model with Dirichlet priors. The BDe metric is expressed as:(2)

where Γ(●) is the Gamma function, n is the number of nodes, r_i _is the arity of node i, q_i _is the arity of π_i _(the parent set of node i), N_ij _is the number of samples where π_i _is in configuration j, N_ijk _is the number of sampler where π_i _is in configuration j and the node has value k and α_ij _and α_ijk _are the prior counts corresponding to N_ij _and N_ijk _respectively. A full derivation of the BDe metric is described elsewhere [19].

In practice, the BDe metric is represented in log-space for computational convenience. Because of this log transformation, all BDe scores in this paper will be negative.

### Calculating the Marginal Likelihood with Missing Data

If we have some missing values or latent variables in the dataset, we can use an altered method for computing the marginal likelihood. Because the likelihood is no longer fully factorizable into the product of the probabilities for each variable, we must calculate the marginal likelihood given every potential completion of the missing data. The simplest method is to marginalize over all possible sets of values for the missing data and take the average. However, this exact enumeration approach is impractical for non-trivial cases and so a heuristic must be used.

In this work we will used a modified Gibbs sampling [19] approach to approximate the marginal likelihood. We alter the method to only sample missing data completions that result in a maximum entropy discretization for all variables. This maximum entropy requirement ensures that p(x_i_) = p(x_j_) for all sets of variables and eliminates bias due to differential discretization for observed and hidden variables.

### Calculating the Marginal Likelihood with Interventions

When calculating the marginal likelihood, data from interventions such as genetic knockouts are handled differently. If a particular data sample is the result of an intervention on a set of variables, the values for those variables no longer depend on their parent sets. They are, instead, arbitrarily set as the result of the intervention. Accordingly, the specific variable samples are ignored when constructing the multinomial table for any node that was intervened upon [23].

### Assessing Model Significance with Bootstrapping based p-values

To evaluate the significance of an MBN prediction, we calculate a p-value for each MBN result using nonparametric bootstrapping [24]. In this approach, we generate a large number of MBN models with template nodes replaced by randomly generated variables with the same discretization schemes as the observed variables. The scores for these MBN models are used as the null model against which MBN scores are compared to determine p-values. Due to the computational time required to score each bootstrap sample, we can only resolve p-values down to .001 and cannot correct for multiple hypothesis testing. We acknowledge that correction for multiple testing would lower the significance of each result.

### Using MBN Templates to Define and Identify Downstream Targets of the Shh Pathway

To determine the downstream targets of the Shh pathway, we created an MBN template based on the canonical pathway as described in literature with a terminal downstream template node as shown in Figure 1(c). Specific MBN models are instantiated from the template by replacing the template node (the candidate target gene) with a specific gene from the dataset. Thus, each instantiated MBN model is a separate hypothesis that can be evaluated against the experimental data.

### Experimental Data

To test our approach, we assembled data from three mouse models with gene knockouts in *Shh*, *Ptch1*, and *Smo *described in more detail elsewhere [25]. Briefly, samples from different embryonic tissues at varying developmental stages were assayed using the U74v2 Affymetrix microarrays to determine the expression profile. Not all combinations of developmental stages and genetic backgrounds were available due to prenatal lethality for *Ptch1 *and *Smo*. Samples assayed include 6-8-somite-stage (approximately 8.5 days post fertilization) with wildtype, *Smo*^-/-^, and *Ptch1*^-/- ^backgrounds; 10-13 somite-stage (approximately 8.75 days post fertilization) with wildtype, *Smo*^-/-^, and *Ptch1*^-/- ^backgrounds; 10.5 days post fertilization embryo samples from head, trunk, and limb bud with wildtype and *Shh*^-/- ^backgrounds. The experimental design is summarized in Table 1.

**Table 1:** Experimental design for gene expression data

*Developmental stage and location*	wt	*Shh*^-/-^	*Ptch1*^-/-^	*Smo*^-/-^
Embryonic day 8.5 whole embryo	3		3	3

Embryonic day 8.75 whole embryo	3		3	3

Embryonic day 10.5 head	4	4		

Embryonic day 10.5 limb bud	6	6		

Embryonic day 10.5 trunk	3	3		

Experimental design for gathering gene expression data from a range of developmental stages, locations, and genetic backgrounds. Columns indicate the genetic background of the mouse model, rows indicate the developmental stage and location of the samples and the numbers within the table cell indicate the number of samples assayed.

### Data Preprocessing

The raw gene expression data (.CEL files) were processed using RMA [26] as implemented in the Bioconductor Affy package [27]. The data were annotated using updated probeset definitions (.CDF files) provided by the Microarray Lab at the University of Michigan [28].

Data were discretized into 3 bins using the Maximum Entropy discretization scheme described above. Because the data contain samples from varying developmental stages and genes with significant expression variation between stages, genes were discretized separately for each tissue type. This discretization scheme allowed the software to more easily identify relevant patterns within a tissue type. Genes were knocked out by altering the sequence to create inactive protein. Thus, for a knockout on a particular gene, the gene's expression was left unaltered but the protein expression was set to 0 and the value was marked as an intervention so it could be handled differently by the scoring procedure.

### MBN Template Analysis

To test the MBN template system using the Shh dataset, we developed a template that matched the topology shown in Figure 1(c). In this template, we search for a downstream target node, GeneX, that responds to the Shh signaling cascade. In this search, we assume that each gene has an equal prior probability of being the target node, and all relationships are scored using the BDe score and a Gibbs sampler, described above.

Using this template, we generated 6299 candidate MBN models -- one for each candidate target gene that showed significant differential expression. Due to the custom CDF annotation, Smo was not present on the chip, thus we modeled the Smo mRNA expression as a hidden node. No protein expression was observed in this experiment, so nodes representing protein concentrations or activities were also modeled as hidden. Rather than selecting the number of iterations for the Gibbs sampler a priori, we calculated the posterior distribution P(M|D) with 150 K iterations, then with 300 K iterations. Although the sampler had largely converged at this point as evidenced by the observation that the top 25 results did not change significantly, we resumed the Gibbs's sampler for the 300 k run and sampled another 300 K iterations for a total of 600 K iterations per MBN model. The computation took approximately 5.6 minutes per MBN for a total of approximately 12 hours on a 48-node compute grid. P-values were calculated with bootstrapping describe above using 1054 MBN models to represent the null distribution. All code and data required to replicate the analysis are included in Additional file 1 [see Additional file 1]. The code can be easily modified to run similar analysis on different dataset using different MBN templates.

In addition, we also tested two alternative Bayesian approaches shown in Figure 1(a) and 1(b). These approaches use topologies that are simpler than the MBN approach in 1(c), but less closely adhere to the known mechanism of the biochemistry of the pathway.

## Results and discussion

In the following sections we show the results from the MBN analysis of the Shh knockout dataset and discuss the biological plausibility for each finding. Next we discuss expected hedgehog pathway target genes that were not identified by the MBN and a comparison with other techniques.

The predicted target genes from the MBN analysis are shown in Table 2. The top scoring hits include many known Shh targets as described below. The top hit, *Gas1*, has been shown to be a negative target of Shh signaling whereas knockout and subsequent gain-of-function studies have shown *Gas1 *to be a positive component of the Shh pathway that acts synergistically with *Ptch1 *to bind the hedgehog ligand [5]. The second target, *Gli1*, is one of the three Gli transcription factors that regulate Shh target genes and is often used as a canonical readout of Shh activity. Shh signaling is known to both induce *Gli1 *expression and also regulate its nuclear accumulation and therefore its activity [29]. *Ptch2 *is a homolog of *Ptch1 *but has a slightly different expression pattern. Like *Ptch1*, *Ptch2 *is known to be transcriptionally modulated by Shh signaling [30]. Knockout studies have shown that Shh signaling is required for expression of *Msx1*, a transcriptional repressor with a putative role in limb-pattern formation [31]. *Foxc2 *and *Foxd1 *have been shown to be upregulated by Shh signaling in null, conditional, and constitutively active Smo mutant backgrounds [32].

**Table 2:** Top 10 results from the MBN analysis (model in Figure 1c).

Gene	Bayesian Score	P-value	Shh Correlation	Ptch1 Correlation	Citations
Gas1 (Growth arrest specific 1)	-226.34	< .001	-0.016	-0.57	[5]

Gli1 (GLI-Kruppel family member)	-226.69	< .001	0.61	0.74	[29]

Ptch2 (Patched homolog 2)	-229.17	< .001	0.54	0.69	[30]

Gtpbp4 (GTP binding protein 4)	-229.44	< .001	-0.28	0.35	

Mig12 (Mid1 interacting protein 1)	-229.61	< .001	0.20	0.61	

Msx1 (Homeo box, msh-like 1)	-229.87	< .001	0.03	-0.56	[31]

Crabp2 (Cellular retinoic acid binding protein II)	-229.97	< .001	-0.05	-0.46	

Has2 (Hyaluronan synthase 2)	-231.03	< .001	-0.08	0.46	

Foxd1 (Forkhead box D1)	-231.61	< .001	0.29	0.43	[32]

Ak1 (Adenylate kinase 1)	-232.22	< .001	0.16	-0.01	

The columns specify the gene name and a short description; the Bayesian score as described in Methods; the resulting p-value for the Bayesian score calculated using a bootstrap method as described in Methods; a linear correlation to the Shh transcript; a linear correlation to the Ptch1 transcript; and a literature citation, if available, that specifies that the gene is indeed a true downstream target of the Shh pathway.

Beyond these known Shh-related genes, some of the highly ranked results appear to be novel putative Shh targets. *Mig12 *binds the Opitz syndrome gene *Mid1 *and the complex is thought to stabilize microtubules [33]. *Mid1 *is repressed by *Shh *[34] and this is partially borne out by our gene expression results which show a positive influence from *Shh *and a negative influence from *Ptch1*. Interestingly though, our analysis shows *Mid1 *to be a poor target. *Mig12 *is not a known Shh target but is a significant result in our analysis and appears to be upregulated by the Shh pathway.

Both cellular retinoic acid binding proteins, *Crabp1 *and *Crabp2*, score well in our analysis but are not known Shh targets. In some developmental processes, retinoic acid (RA) has been shown to work synergistically with Shh to create the zone of polarizing activity, [7] for example, and in adult pluripotent stem cells [8]. Crabp binds RA with high affinity and is thought to spatially modulate the effect of RA by adjusting the concentration of RA reaching the nucleus [35]. One interpretation of the MBN result is that that Shh modulates the effects of RA by regulating Crabp.

### Known Shh targets not identified by the MBN

**Figure 2:**
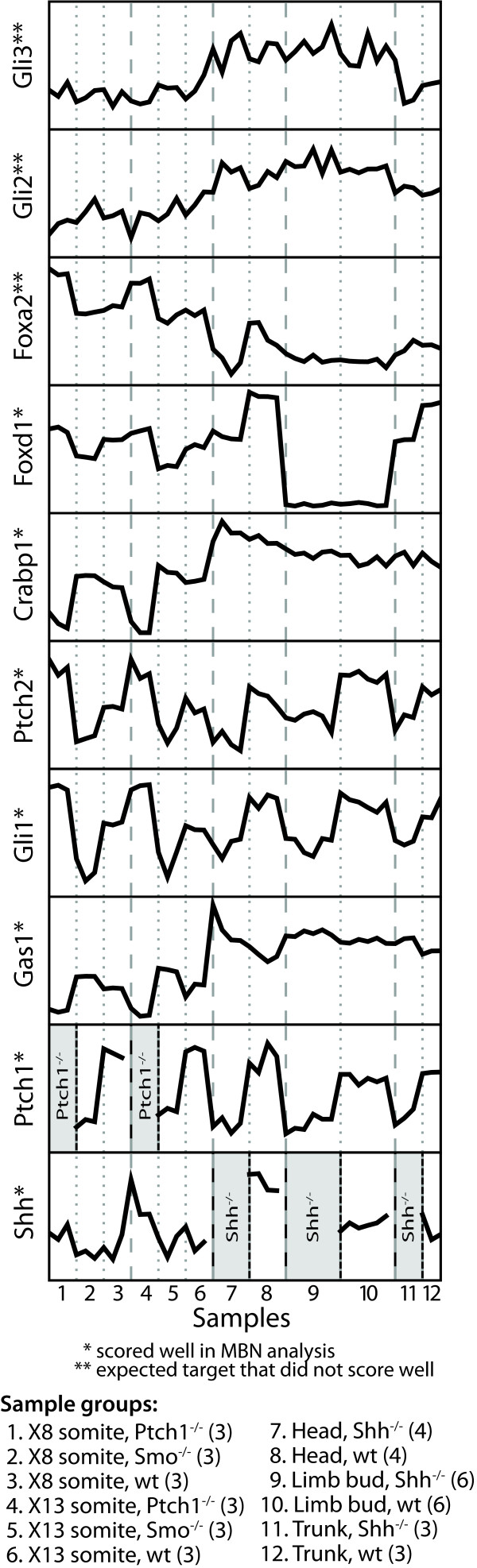
**Comparing the relative gene expression profile of 8 selected putative targets**. From these expression patterns one can see that the profiles for *Gas1 *and *Gli1 *closely follow changes in *Shh *and *Ptch1 *expression. *Gli2 *and *Gli3 *show higher activation in the adult tissues as compared to the somite samples but, even in the adult tissues, their expression pattern does not correspond to the knockout state of *Shh*. *Foxa2 *expression shows a strong response to the *Ptch1 *knockouts in the somite samples and with *Shh *knockouts in the adult head tissues but there is no pattern in the adult limb and trunk tissues. While the patterns in *Gli2*, *Gli3*, *Foxa2 *are significant, the patterns either do not coincide with patterns in the early steps of the Shh pathway (*Shh*, *Ptch1, Smo*) indicating that there may be other genes involved in their regulatory control or the patterns do not hold over all tissues. Accordingly, those genes do not score highly in the MBN method.

Our results, however, do fail to identify some known and expected targets. For example, one might expect the genes *Foxa2*, *Gli2*, *Gli3*, and *Ihh *to be identified by the MBN as these are often listed as targets of the Shh pathway, but in our analysis they did not score near the top. The MBN template used in this analysis (Figure 1) detects relationships between a candidate target and *Shh *and *Ptch1*, constrained by the information bottleneck introduced by the hidden protein nodes. When we examine the relationship between the expected but poorly scoring genes and *Shh *and *Ptch1 *(Figure 2), we find no consistent pattern of differential expression. It is clear that the expression does not change significantly or consistently with the pattern of change for *Shh *or *Ptch1*. Genes identified as targets by the MBN such as *Gas1*, *Gli1*, *Ptch2*, *Crabp1 *and *Foxd1*, show clear relationships that can be assessed by eye.

### Comparison of MBN Analysis and non-Bayesian Bioinformatics Techniques

The results we obtain using MBN are different than those from standard techniques for determining downstream pathway targets. Whereas clustering, differential expression and significance testing are, in general, unable to identify nonlinear and multimodal relationships, Bayesian methods such as MBN do not have this limitation. Bayesian methods learn based on complex patterns of change rather than the magnitude of change or a simple linear correlation.

When we use differential expression as an indicator of significance, for example, we find none of the canonical targets near the top of the list [see Additional file 2]. *Gli1*, the commonly used indicator of Shh activity, ranks 629^th ^in terms of fold-change, but 2^nd ^with respect to MBN analysis. This difference is expected, however, because only small changes in gene expression are required to modulate the activities of transcription factors and pathway components that participate in feedback loops with the Shh ligand. We obtain slightly better but still poor results when identifying targets based on a linear correlation to either *Shh *or *Ptch1 *gene expression. This difficulty with clustering can be seen in Table 2. Clustering methods suffer from similar failures as linear correlation and fail to take into account known information about the initial steps of the pathway.

### Comparison of MBN and BN modeling of the Shh Pathway

To isolate the effect of the MBN template over more standard BN approaches, we ran a direct comparison between the approaches using the three topologies shown in Figure 1. The simplest translation of the Shh pathway, shown in Figure 1(a), is to interpret the qualitative diagram as a BN and replace all arrows with a Bayesian edge to produce a sequential model. Although the topology looks most similar to a biochemical diagram, for a Bayesian network the topology indicates that only Smo influences the target gene. An alternative topology is shown in Figure 1(b). In this parallel topology, the target gene is influenced by all of the genes in the pathway. However, this topology does not capture the unobserved proteomic effects, nor does the topology distinguish between the relative position of *Shh, Ptch*, and *Smo *in the signaling pathway

The results for the three topologies depicted in Figures 1(a), 1(b) and 1(c) are shown in Tables 3, 4 and 2 respectively. When we compare the results, we find that the target genes predicted by the MBN topology (Figure 1(c)) best match what is known about the pathway. If we compare the number of known targets identified in the top 10 targets predicted by each method we find the MBN predicts 5/10, the parallel topology predicts 4/10, and the sequential topology predicts 2/10. In the MBN results, Gas1, Gli1 and Ptch2 rank 1^st^, 2^nd^, and 3^rd ^respectively and are all well-known Shh targets, while in the sequential model these genes rank 11^th^, 2^nd ^and 3^rd ^respectively, and in the parallel model these genes rank 24^th^, 20^th ^and 21^st ^respectively. One reason for this discrepancy is that the BN models impose fewer constraints on the candidate gene which would produce more false positives -- a result in line with what we see. The full lists of target genes for each topology is provided in Additional file 2.

**Table 3:** Top 10 results from the sequential BN analysis (model in Figure 1a).

Gene	Bayesian Score	Citations
Ak1 (Adenylate kinase 1)	-80.30	

Ubc (Ubiquitin C)	-81.84	

Pdcd4 (Programmed cell death 4)	-81.90	

Nckap1 (NCK-associated protein 1)	-81.90	

Crabp2 (Cellular retinoic acid binding protein II)	-82.07	

Ntn1 (Netrin 1)	-82.60	[37]

Oprs1 (Opioid receptor, sigma 1)	-82.70	

Fgf8 (Fibroblast growth factor 8)	-82.84	[6]

Nme6 (Expressed in non-metastatic cells 6, protein)	-83.10	

Gtf3c5 (General transcription factor IIIC, polypeptide 5)	-83.10	

The columns specify the gene name and short description; the Bayesian score as described in Methods; and a literature citation, if available, that specifies that the gene in indeed a true downstream target of the Shh pathway.

**Table 4:** Top 10 results from the parallel BN analysis (model in Figure 1b).

Gene	Bayesian Score	Citations
Foxc2 (Forkhead box C2)	-115.16	[32]

Gli1 (GLI-Kruppel family member GLI1)	-116.19	[29]

Ntn1 (Netrin 1)	-117.24	[37]

Ptch2 (Patched homolog 2)	-117.24	[30]

Gspt1 (G1 to S phase transition 1)	-117.39	

Mid1ip1 (Mid1 interacting protein 1)	-117.39	

B4galt3 (UDP-Gal:betaGlcNAc beta 1,4-galactosyltransferase, polypeptide 3)	-117.51	

Cklfsf8 (Chemokine-like factor super family 8)	-118.05	

Ptprz1 (Protein tyrosine phosphatase, receptor type Z, polypeptide 1)	-118.20	

Dctn3 (Dynactin 3)	-118.42	

The columns are identical to those in table 3.

### Extension to larger MBN templates

While we used a small template pathway in this work as a proof of concept, the method can be applied to larger pathways in a similar way. However, large numbers of hidden nodes increase the computational requirements and may make the analysis intractable using the Gibbs sampler used in this work. The MBN template algorithm's runtime complexity is O(nm^2^h^2^) where n is the number of candidate genes, m in the number of data samples and h is the number of hidden or latent variables. Because the limiting step is the Gibbs sampling's O(m^2^h^2^) runtime complexity, we could use a more complex but efficient heuristic sampling method such as variational learning [36]. As currently implemented, MBN templates should be limited to models with a small number of hidden nodes.

### Extension of MBNs to other data types

Although this study focused on gene expression data, the MBN template method can be used with any type of observed data that can be represented in the pathway. If a molecular entity can be represented in an MBN template while being faithful to the underlying biochemical mechanisms, data regarding the molecule's concentration or activity can be used. Possible other measurements that could be used with a similar MBN approach include protein expression, kinase activity, and miRNA expression. Furthermore, MBN templates can be used to integrate observations for multiple data types assuming that the measurements are made on a common set of samples.

## Conclusion

We have shown how MBNs can be used to integrate gene expression data and known topological information to uncover mechanistic details about complex pathways. Although we have shown only one example with finding downstream targets of the Shh pathway, a similar approach could be used on other pathway topologies, data types, and to identify targets at different locations in the pathway. In this example, the MBN method provided better target predictions than other methods, due in part because the topology assumed by the MBN is closer to the known biochemical mechanism.

## List of Abbreviations

**MBN**: mechanistic Bayesian network; **BN: **Bayesian network

## Authors' contributions

AS Designed the MBN software, carried out the analysis, and wrote much of the paper. TT gathered the gene expression data and commented on the results. APM Designed the experiment, oversaw the data collection process, and commented on the results. PJW Designed the MBN approach, guided the analysis, and edited the paper. All authors have read and approved the final manuscript.

## Supplementary Material

Additional File 1 **Source code and data**. Contains all the code and data required to replicate the analysis. The scripts use PEBL's distributed computing features to run analysis over a variety of distributed computing platforms. Additionally, the scripts are easy to modify to run similar analysis with different datasets and MBN templates. The README.txt file contains further information about using and modifying the scripts. Click here for file

Additional File 2 **Complete list of predicted Shh targets**. Excel file showing the complete target lists ranked by their fit within the MBN and BN frameworks (given all three models in Figure 1). The file also contains results for two smaller studies using the Affymetrix 430A and 430B chips. Click here for file
