# Supplementary material for: Using mechanistic Bayesian networks to identify downstream targets of the Sonic Hedgehog pathway
Source: BMC Bioinformatics. 2009 Dec 18;10:433. doi: 10.1186/1471-2105-10-433 (PMC3087349; doi:10.1186/1471-2105-10-433)
Supplement: Additional File 2 — Complete list of predicted Shh targets. Excel file showing the complete target lists ranked by their fit within the MBN and BN frameworks (given all three models in Figure 1). The file also contains results for two smaller studies using the Affymetrix 430A and 430B chips. [file 1471-2105-10-433-S2.TGZ › shh.targets.code/pebl-1.01.tar.gz!/pebl-1.01/docs/src/static/tutorial/example1-result/index.html]

Pebl Results


- Statistics
- Top Scoring Networks
- Consensus Networks

Run Statistics

|  |  |  |
| --- | --- | --- |
| Run | Host | Runtime (in mins) |

|  |  |
| --- | --- |
|  | Rank: |

Use common layout for all networks

Threshold for Consensus Network: 
10%
20%
30%
40%
50%
60%
70%
80%
90%
